# Application of Bland-Altman Method in Comparing Transrectal and Transabdominal Ultrasonography for Estimating Prostate Volume

**Published:** 2015-01

**Authors:** Ali Babaei Jandaghi, Maryam Shakiba, Hamidreza Nasseh, Yaser Korouji, Samaneh Esmaeili, Ali Akbar Khadem Maboudi, Ali Khorshidi

**Affiliations:** 1 Department of Radiology, Guilan University of Medical Sciences, Rasht, Iran;; 2 Department of Epidemiology, Shahid Beheshti University of Medical Science, Tehran, Iran;; 3 Urology Research Center, Guilan University of Medical Sciences, Rasht, Iran;; 4 Department of Biostatistics, Shahid Beheshti University of Medical Science, Tehran, Iran

**Keywords:** Prostate, Ultrasonography, Cross-sectional studiy, Bland-Altman approach

## Abstract

**Background:**

Estimating prostate volume using less invasive transabdominal ultrasonography (TAUS) instead of transrectal ultrasonography (TRUS) is of interest in terms of identifying their agreement level. Previous reports on this subject, applied general correlation coefficient as the level of agreement. This study uses Bland-Altman method to quantify TAUS and TRUS agreement on estimating prostate volume.

**Methods:**

Total prostate gland volume of 40 patients with signs and symptoms of benign prostatic hyperplasia were measured using TAUS and TRUS. The study was carried out at the Urology Research Center, Razi Hospital, Guilan University of Medical Sciences (Rasht, Iran) from March to October 2010. Both methods were performed in one session by the same experienced radiologist. Data were analyzed using Pearson correlation coefficient and Bland-Altman method.

**Results:**

Total prostate volume estimated by TAUS and TRUS were 50.30±23 and 50.73±24.6 mL, respectively. The limits of agreement for the total prostate volume were -6.86/9.84 that was larger than predefined clinical acceptable margin of 5 mL.

**Conclusion:**

There is a lack of agreement between TAUS and TRUS for estimating the total prostate volume. It is not recommended to apply TAUS instead of TRUS for estimating prostate volume.

## Introduction

One of the tools used in the assessment and evaluation of the anatomy of the prostate (both in benign and malignant diseases of the prostate) is sonographic imaging. The current approach for diagnosing prostate volumes is through transrectal ultrasonography (TRUS). Although this approach is easy-to-use and less expensive than other imaging methods, but often it cause some discomfort for the patients. However, there are contraindications to TRUS (e.g. Mile’s operation) after which patients are likely to have persistent hematuria for up to 3 to 7 days after the procedure.^1^ Alternative approach to TRUS is using transabdominal ultrasonography (TAUS). Previous studies reported excellent correlation between TRUS and TAUS,^2^^-^^5^ despite certain discrepancies in the results. Kim et al. found a strong correlation only for experienced examiners.^6^ In patients with BPH, Stravodimos et al. found TRUS as a more accurate method in predicting adenoma volume than TAUS.^7^

Almost all past studies compared the two measurement techniques using common statistical approaches, e.g. correlation coefficients or paired *t* test. These classical methods for measuring agreement suffer from many limitations since the correlation coefficient depends on the range of true values in the data and only measures the strength of linear association between variables.^8^ *T* test based on comparing means, is related to each individual data and the power of the study.^8^^,^^9^ Because of these misleading approaches, Bland and Altman favored a different statistical method for assessing agreement between two methods of measurement. Their innovation proposed a visual observation of difference between two methods against their mean. If there is no clinical importance in the reported limits of agreement (i.e. mean difference±2standard deviation(SD)) between the two methods, they can be used interchangeably.^9^ This method is the most and still the best technique used to quantify agreement between the two measurements.

Due to the limitation of TRUS, such as cost and causing discomfort to patients, replacing it with TAUS that is cheaper, more convenient, and safer for the patient is desirable. To replace a new method with an established one, two methods that cannot be regarded as the true value of the quantity being measured, should be compared using agreement criteria and should agree sufficiently well.^9^ Hence, the objective of this study is to determine the agreement between TAUS and TRUS. Considering limitation of past studies in evaluating an agreement, this study aimed to compare TRUS and TAUS using Bland-Altman approach to define a priori to examine their agreement.

## Materials and Methods

*Study Sample*

This cross sectional survey was performed on patients with benign prostatic hyperplasia (BPH) referred to the Urology Research Center, Razi Hospital (from March to October 2010). All patients with discomfort during urination and palpable prostate on clinical examination by the urologist were included in the study. Patients with suspected acute prostatitis, prior open prostate surgery, or TURP as well as those disqualified for transrectal sonography were excluded from the study. Informed consent was obtained from patients and the study protocol was approved by the Research Committee of Guilan University of Medical Science.

*Measurement Procedure*

Prostate volume was measured by transabdominal suprapubic sonography followed by transrectal sonography in patients with semi-filled bladder. Both methods were performed by an experienced radiologist and the service was offered free of charge. The sonography was performed with Ultrasonix-OP-01 (Canada, 2005) model system. We used 2.5-5 MHz curved convexity probe for transabdominal sonography and 5-9 MHz endocavitary probe was used for the transrectal sonography. Meanwhile, three dimensions; anteroposterior, transverse, craniocaudal, and prostate volumes were calculated in millimeter. 

*Statistical Method*

The prostate volume was calculated according to the measured indices as described below:

(Anteroposterior diameter×Transverse diameter×Craniocaudal diameter×0.52)

Considering a correlation coefficient of 0.5 with 95% confidence level and 80% power, a total of 40 patients was estimated as the sample size. 

Bland-Altman analysis was used to compare the two measurement techniques. For each dimension, the average of TAUS and TRUS were calculated and then plotted against the difference of the two measurements. Horizontal lines were drawn at the mean difference and at the limits of agreement, which are defined as the mean difference±1.96 times the standard deviation of the differences. Normality distribution of differences was assessed by a histogram. Then the mean of difference and limits of agreement were drawn. For clinical purposes, a difference not exceeding 5 mL between the two measurements was defined as a priori and limits of agreement were compared to it. Data were presented as mean±SD. 

## Results

**Figure 1:**
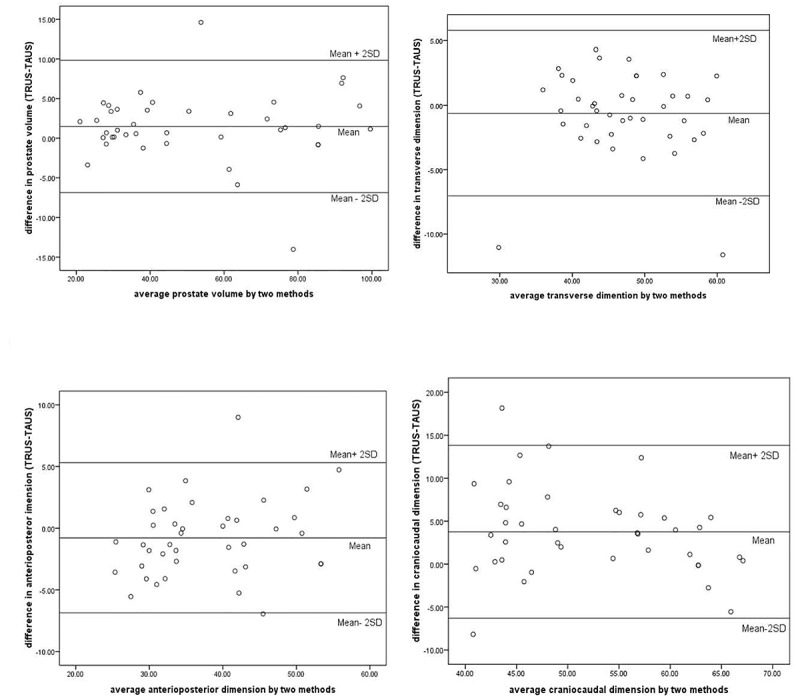
Bland-Altman analysis for prostate volume

Forty consecutive patients (age range, 40-87 years; mean, 66.9±10 years) participated in the study. Patients’ prostate volume was measured by two separate sonographic methods. The mean total prostate volume of all patients estimated by transabdominal sonography was 50.30±23.9 mL while mean prostate volume measurement estimated by transrectal sonography was 50.73±24.6 mL. The lowest and highest total prostate volume measurements of our study population was 21.37±19.9 mL and 100.15±99 mL, respectively. Table 1 illustrates patients’ prostate dimension measured by the two methods. Figure 1, demonstrates Bland-Altman plot. There is no relationship between the difference and the level of measurement in either plots. Therefore, reporting the limit of agreement is valid.

**Table 1:** Comparison of Transabdominal and Transrectal measurement of patients’ prostate dimension

	**Transverse** **mean±SD**	**Anterioposterior** **mean±SD**	**Craniocaudal** **mean±SD**	**Total** **mean±SD**
Transrectal	46.86±7.3	37.48±8.91	54.09±8.36	52.56±24.54
Transabdominal	47.49±7.5	38.27±8.24	50.33±9.53	51.07±24.57
Pearson correlation	0.90*	0.94*	0.84*	0.99*

*Significant at P<0.001

After calculating the mean difference (d) and the standard deviation of the difference (s) based on Bland-Altman analysis, we would expect most of the differences to lie between the limit of agreement (d-2s and d+2s). If the resultant differences within d±2s would not be clinically important, we could use the two measurement methods interchangeably. Defining a priori not exceed than 5 mL, the limits of agreement in all three dimensions are much larger than priori and unacceptable for clinical purposes (table 2 and figure 1). The total prostate volume measurement made by TAUS would be between 6.86 mL less and 9.84 mL more than the measurement made by TRUS, which is wider than the predefined priori. Therefore, according to the Bland-Altman method, there was a lack of agreement between the two methods, despite an excellent degree of correlation in all three dimensions (table 1).

**Table 2:** The mean differences and limits of agreement of prostate dimension measured by TAUS and TRUS

	**Mean difference**	**Limits of agreement**
Transverse	-0.63	-7.04/5.78
Anterioposterior	-0.78	-6.87/5.31
Craniocaudal	3.76	-6.31/13.83
Total	1.49	-6.86/9.84

TRUS: Transrectal ultrasonography, TAUS: Transabdominal ultrasonography

## Discussion

Medical technology assessments are often compared with established techniques to be used interchangeably.^10^^,^^11^ In this sense, neither methods cannot be regarded as true value and their agreement is the matter to be known.^12^ Historically, in method comparison studies, some familiar statistical methods such as correlation coefficient, paired *t* test or Intra-Class Correlation Coefficient (ICC) had been used that were obviously inappropriate to answer the question and their limitation have been described.^3^ Bland and Altman proposed a different statistical method based on visual observation and defining limits of agreement for assessing comparison between two methods.^12^ This method is currently the most commonly used approach for measuring agreement.

According to Bland-Altman approach, the present study revealed that there is a lack of agreement between transrectal and transabdominal sonography for the measurement of the total prostate volume. Previous studies quantifying agreement with correlation coefficient such as Ozden et al. (r=0.94),^2^ Prassopoulos et al. (r=0.95),^3^ and Chung et al. (r=0.84)^4^ showed a strong correlation. However, Gloi et al. used Bland-Altman method to determine agreement between the computed tomography and ultrasound for the measurement prostate volume. The level of agreement between those two methods, which were strongly correlated, was not approved by Bland-Altman method.^13^

Choosing the correct statistical method for examining the agreement between two continues variable is of great importance. Classical approaches such as correlation coefficient and linear regression are misleading.^9^^,^^12^ Pearson correlation measures the strength of a relation between two variables, not the agreement between them. It also depends on the range of the true quantity in the sample, if this is wide, the correlation will be greater than when it is narrow.^12^ In this study, the range of prostate volume by the two methods varies from less than 30 mL to more than 60 mL and the magnitude of the correlation coefficient varies depending on the range of the prostate volume. It is also found that there is a strong correlation between the two methods (r measure ranging from 0.84-0.99) but the limits of agreement were larger than the predefined clinical margin.

For replacing a new diagnostic method with the old one, the primary question is that if the two methods agree sufficiently to be replaced with each other.^9^ Bland and Altman put forward an alternative method based on the “limits of agreement” technique. If the “limits of agreement” (mean difference±2standard deviation(SD)) are not clinically important, then one can use two measurements interchangeably. Many previous authors of method comparison studies introduced Bland-Altman analysis as the best method for measuring agreement.^8^^,^^14^^,^^15^

As stated before, the correlation coefficient is not a measure of agreement; it is a measure of association.^12^ This study revealed a significant high correlation coefficient that was not approved by the Bland-Altman method. The correlation coefficient depends on both the variation between individuals and the variation within individuals. It will therefore partly depend on the choice of subjects. If there is a high variation between individuals compared to the measurement error, the correlation will be high and vice versa.^12^ Therefore, we cannot conclude that the two methods can be used interchangeably if there is a high correlation between them.^16^ In this study, the correlation coefficient ranges from 0.99 for the total gland size to 0.84 for craniocaudal dimension which can correspond to a higher variation in the total gland size (SD=24.57) than craniocaudal dimension (SD=8.36).

Some other studies compared the mean of readings from two instruments using *t* test or Mann-Whitney U test to report their agreement.^17^^-^^19^ An insignificant difference in paired *t* test result does not provide evidence for agreement. The mean value is affected by the value of individual data which in turn can be influenced by extremely large or extremely small values.^7^ Thus, it is possible to observe almost the same value for the mean of the two methods but there is a large variation in the distribution of data.

A major assumption in “quantitative method comparisons” studies is that the limits of agreement should be valid across the whole range of values.^9^ Based on the assumptions of (i) normal distribution of difference and (ii) no relationship between differences, the level of measurement should be assessed before determining the limits of agreement. In this study, both assumptions were evaluated and confirmed. The limits of agreement need to define a priori in the methods to be compared with it. However, few studies report on such definition.^15^ Previous study by Chung et al. did not report the limits of agreement and no comparisons were made with respect to priori.^5^ This study defined a priori of ±5 mL to compared the limit of agreement.

In this study, transabdominal sonography was performed on patients with semi-filled bladder, since overfilled bladder compromised circulation and pressure on prostate and leads to lack of correct recognition of the prostate size. 

Our study had some limitations; namely, both measurement methods (TRUS and TAUS) were performed by one experienced radiologist but at the same time. Therefore, some measurement bias due to pre-knowledge of measures may ensue. To overcome this bias, at least two patients’ prostate was measured in each session, two techniques were performed at different times, and the records of each measurement were blinded from the radiologist. Another limitation was related to the concept of agreement. In measuring agreement, the two methods that, neither of which revealed the true values were compared such that the accuracy of the newer method could not be established. However, in the calibration studies, the new method is compared with a precise one as gold standard. This topic was not the objective, but strongly recommended in the context of further research. 

## Conclusion

Based on the findings of the current study, transabdominal ultrasonography did not agree sufficiently with transrectal ultrasonography. Therefore, it cannot replace TRUS for measuring prostate volume. Since TRUS is not the gold standard for estimating prostate volume and measuring the agreement between TRUS and TAUS is still debatable, further research is recommended to determine calibration of TAUS with a more accurate gold standard. 
